# Food Insecurity—A Risk Factor for HIV Infection

**DOI:** 10.1371/journal.pmed.0040301

**Published:** 2007-10-23

**Authors:** Nigel Rollins

## Abstract

Nigel Rollins discusses a new study showing that food insufficiency is a risk factor for increased sexual risk-taking in women in Botswana and Swaziland.

## Nutrition, Food, and HIV

HIV and nutrition are linked in at least two important ways. First, the nutritional consequences of HIV have been obvious from the earliest reports of the epidemic in Africa. Patients suffering from the infection in Uganda were said to have “slim disease” [1]. More than 25 years later, we are still grappling with the mechanisms by which HIV causes wasting and defining the macronutrient and micronutrient requirements of adults and children infected with the virus [2]. The World Health Organization (WHO) recommends that energy requirements of HIV-infected individuals increase by about 10% from the time of infection and by 20%–30% when chronic opportunistic infections or HIV-specific conditions are present [3,4]. The WHO also recommends that HIV-infected patients should be assured of at least one recommended daily allowance of most vitamins. In the absence of an adequate diet, this often means that HIV care and treatment programmes must supply multiple micronutrient preparations [3,5].

The second important link between HIV and nutrition is the growing realization that food insecurity (lacking adequate food supply to meet daily needs) may increase HIV risk transmission behaviours and susceptibility to HIV once exposed. Observational studies, for example, have reported an association between communities suffering poor food security and HIV transmission [6]. Poverty, and the concern for dependents, can drive individual behaviour in ways that place health and safety at risk. However, little is known about the specific mechanisms by which food insecurity influences risk-taking behaviour and consequent vulnerability to HIV transmission.

**Linked Research Article**

This Perspective discusses the following new study published in *PLoS Medicine:*

Weiser SD, Leiter K, Bangsberg DR, Butler LM, Percy-de Korte F, et al. (2007) Food Insufficiency Is Associated with High-Risk Sexual Behavior among Women in Botswana and Swaziland. PLoS Med 4(10): e260. doi:10.1371/journal.pmed.0040260

In a cross-sectional study, Sheri Weiser and colleagues found that food insufficiency was an important risk factor for increased sexual risk-taking in women in Botswana and Swaziland.

## A New Study on Food Insecurity and HIV Risk Behaviour

In a new cross-sectional study published in *PLoS Medicine*, Sheri Weiser and colleagues collected data on both food security and HIV risk behaviour from population-based samples from five districts in Botswana and all four districts of Swaziland. In total, 2,051 adults were interviewed [7]. The study participants were asked about the adequacy of their food intake over the preceding 12 months, and these data were related to condom usage, sex exchange, and other HIV risk behaviour such as having multiple partners. (For women, sex exchange was defined as exchanging sex for money, food, or other resources over the previous 12 months and for men this was defined as paying for or providing resources for sex over the previous 12 months.) Gender equity was explored through questions assessing aspects of sexual relationships.

The results were striking. Of all the study participants, 32% of women and 22% of men had experienced food insufficiency in the preceding 12 months. Food insufficiency was associated with increased HIV risk behaviour, and this association was much more marked in women than men. Risk behaviour included inconsistent condom use, sex exchange, increased intergenerational sex, and lack of control over sexual relationships.

This is an important study, with major implications for policy makers, although there are some methodological weaknesses that limit how the data can be analysed and interpreted. One year is a long period for accurate recall of food sufficiency and the details of sexual relationships. Categorising household income below and above national averages is a crude indicator of income and does not reflect access to money and economic dependency within the household. And no objective measures of nutritional status were included in the study to substantiate nutritional vulnerability. Nevertheless, the message of the study is clear: in the absence of adequate food for oneself or one's family, individuals will forfeit long-term personal safety to survive today.

## Implications

For programme planners, these findings provide an additional rationale, even obligation, to consider hunger alleviation as a central component of HIV prevention programmes. In poverty-stricken communities, the incentive of reducing HIV risk behaviour should be an added reason for national governments and international agencies to invest in reducing hunger by improving infrastructure and development—as outlined in the Millennium Development Goals 1, 4, 5, and 6 (reducing extreme poverty and hunger, reducing child and maternal mortality, and preventing the spread of HIV/AIDS). Accordingly, the Global Fund to Fight AIDS, Tuberculosis and Malaria will need to consider how it deals with requests to provide food, as part of HIV prevention strategies, to populations known to be at high risk of food insecurity and, therefore, at risk of HIV infection.

Researchers designing behaviour change strategies to reduce HIV risk may also need to contemplate ways of improving food security in vulnerable groups. Failure to do so may limit the effectiveness of other interventions aimed at improving seemingly unrelated outcomes. Similar to providing condoms and counselling to study participants in prevention trials, researchers may also be ethically obliged to provide support to alleviate acute food insecurity when identified.

Reducing food insecurity is, however, complex, especially in the context of HIV. In Africa, where HIV incidence is high and food insecurity widespread, the logic of linking initiatives is clear. In other regions such as Asia, where HIV prevalence is relatively low, targeted interventions to reduce hunger in order to reduce HIV transmission amidst widespread food insecurity could be practically and ethically difficult to implement. For example, would it be unethical to provide food for the purpose of reducing HIV transmission risk as a way of reducing commercial sex work in poor, food-insecure regions or communities?

## Future Research

The magnitude of the increased risk of HIV infection faced by poor and vulnerable women in areas of food insecurity needs to be better quantified, using methodologies that define clearly environmental influences such as drought, food production, and local cash flows as well as the personal interactions of women and men in such communities. Prevention interventions must address both the physical needs of hungry people as well as the autonomy that young women need to exercise their choices. Could conditional grants, for example, giving adolescent girls monthly allowances, be used to reduce gender pressures and HIV incidence in communities with high unemployment and teenage pregnancy rates? Would the economics of hunger reduction satisfy the donors focused on HIV?

Sheri Weiser et al. remind us that recognising and reporting the obvious is not always commonplace. Ignoring such basic issues as food or hunger could be a major stumbling block to HIV prevention strategies.

## Abbreviations

WHO: World Health Organization
